# 

**Published:** 1842-04-16

**Authors:** 


### CONTENTS OF No. 16.

Dr. Isaac Parrish on the Use of the Iodide of Potassium in Ophthalmic Diseases, with cases, . . . . . . . . . . . 241
Dr. J. W. Ash's Case of Latent Typhoid Fever, . . . . : 243
BIBLIOGRAPHICAL NOTICES: Prof. J. B. Beck's Observations on the Signs of Live and Still Birth, . . . . . 245
Dr. Stewardson on Remittent Fever, . . . . . 248
ANALECTA: Dr. Fitzpatrick on Scarlatina, . . . . . . 253
Case of Spontaneous Dislocation and Anchylosis of the First and Second Cervical Vertebræ, . . . . . . 238

